# Correction: A lexicon-based diachronic comparison of emotions and sentiments in literary translation: A case study of five Chinese versions of David Copperfield

**DOI:** 10.1371/journal.pone.0315963

**Published:** 2024-12-12

**Authors:** Yan Li, Muhammad Afzaal, Yixin Yin

There is an error in affiliation 2 for author Yan Li. The correct affiliation 2 is: Graduate School of Translation and Interpretation, Beijing Foreign Studies University, Beijing City, China.
