# Supplementary material for: A lexicon-based diachronic comparison of emotions and sentiments in literary translation: A case study of five Chinese versions of David Copperfield
Source: PLoS One. 2024 Feb 13;19(2):e0297101. doi: 10.1371/journal.pone.0297101 (PMC10863854; doi:10.1371/journal.pone.0297101)
Supplement: S1 Appendix — (DOCX) [file pone.0297101.s001.docx]

Appendix I Top 30 High-Frequency Positive Words in Five Translations

| sequence | Lin's | frequency | Xu's | frequency | Dong's | frequency | Zhang's | frequency | Zhuang's | frequency |
| --- | --- | --- | --- | --- | --- | --- | --- | --- | --- | --- |
| 1 | 孺子 | 134 | 希望 | 352 | 相信 | 641 | 不错 | 417 | 希望 | 403 |
| 2 | 亲吻 | 36 | 相信 | 300 | 一定 | 438 | 一定 | 410 | 高兴 | 375 |
| 3 | 大笑 | 34 | 一定 | 289 | 希望 | 362 | 高兴 | 317 | 一定 | 351 |
| 4 | 精神 | 24 | 朋友 | 244 | 喜欢 | 338 | 相信 | 292 | 朋友 | 250 |
| 5 | 鞠躬 | 24 | 高兴 | 201 | 朋友 | 252 | 希望 | 254 | 喜欢 | 244 |
| 6 | 入时 | 23 | 愿意 | 200 | 高兴 | 205 | 喜欢 | 228 | 愿意 | 221 |
| 7 | 学问 | 22 | 快乐 | 192 | 快活 | 175 | 朋友 | 225 | 相信 | 191 |
| 8 | 爱情 | 22 | 喜欢 | 191 | 愉快 | 168 | 愿意 | 164 | 愉快 | 147 |
| 9 | 不期 | 21 | 神气 | 123 | 可爱 | 163 | 快活 | 163 | 肯定 | 146 |
| 10 | 回首 | 20 | 可爱 | 113 | 必须 | 152 | 神气 | 115 | 漂亮 | 140 |
| 11 | 时尚 | 20 | 愉快 | 103 | 神气 | 136 | 精神 | 97 | 认真 | 111 |
| 12 | 朋友 | 17 | 注视 | 99 | 最好 | 108 | 尽力 | 94 | 热情 | 104 |
|  | 得意 | 17 |  |  |  |  |  |  | 最好 | 104 |
|  | 坚定 | 16 |  |  |  |  |  |  | 快活 | 104 |
| 13 | 回顾 | 14 | 自然 | 98 | 爱情 | 103 | 大笑 | 78 | 回想 | 102 |
|  | 自信 | 14 |  |  | 愿意 | 103 |  |  |  |  |
| 14 | 徐徐 | 12 | 微笑 | 97 | 精神 | 93 | 温柔 | 76 | 幸福 | 99 |
|  | 祝福 | 12 | 美丽 | 97 |  |  |  |  |  |  |
| 15 | 感激 | 11 | 爱情 | 94 | 幸福 | 91 | 真正 | 75 | 自然 | 85 |
|  | 生趣 | 11 |  |  |  |  |  |  | 可爱 | 85 |
| 16 | 如故 | 10 | 最好 | 90 | 信任 | 89 | 舒服 | 74 | 精神 | 84 |
| 17 | 自觉 | 10 | 欣喜 | 86 | 奇怪 | 88 | 感激 | 72 | 温柔 | 79 |
| 18 | 求婚 | 10 | 真正 | 85 | 满意 | 87 | 安静 | 70 | 舒服 | 76 |
|  | 小儿 | 10 |  |  | 快乐 | 87 |  |  | 严肃 | 76 |
| 19 | 聪明 | 10 | 舒服 | 81 | 帮助 | 86 | 自然 | 69 | 不错 | 73 |
| 20 | 飞越 | 10 | 温柔 | 79 | 微笑 | 83 | 快乐 | 65 | 原谅 | 69 |
| 21 | 阳光 | 10 | 必须 | 78 | 恢复 | 78 | 得意 | 63 | 善良 | 67 |
|  | 微笑 | 10 |  |  |  |  | 微笑 | 63 | 疼爱 | 67 |
| 22 | 美丽 | 9 | 快活 | 73 | 热情 | 77 | 回忆 | 58 | 满意 | 63 |
|  | 持家 | 9 | 可喜 | 73 |  |  | 疼爱 | 58 |  |  |
| 23 | 自然 | 9 | 感激 | 70 | 平静 | 76 | 确实 | 57 | 微笑 | 59 |
|  | 希望 | 9 |  |  |  |  | 帮助 | 57 | 欢迎 | 59 |
| 24 | 名誉 | 9 | 漂亮 | 69 | 舒服 | 75 | 美丽 | 56 | 感激 | 57 |
|  | 安适 | 9 | 安静 | 69 |  |  |  |  |  |  |
| 25 | 挺立 | 9 | 精神 | 66 | 安静 | 74 | 坚定 | 55 | 奇怪 | 56 |
|  | 恭谨 | 9 | 坚定 | 66 |  |  |  |  | 平静 | 56 |
| 26 | 交谊 | 9 | 宝贝 | 65 | 开心 | 72 | 热烈 | 53 | 必须 | 53 |
|  | 乐趣 | 8 |  |  |  |  | 爱慕 | 53 |  |  |
| 27 | 君子 | 8 | 乐于 | 62 | 满足 | 71 | 了不起 | 52 | 安慰 | 52 |
|  | 注视 | 8 |  |  |  |  | 幸福 | 52 |  |  |
| 28 | 倾听 | 8 | 人儿 | 60 | 自然 | 70 | 恢复 | 51 | 重要 | 49 |
|  | 释然 | 8 | 倾听 | 60 |  |  |  |  |  |  |
| 29 | 必得 | 8 | 确定 | 58 | 坚定 | 68 | 漂亮 | 49 | 保佑 | 48 |
|  | 平安 | 8 |  |  | 欢喜 | 68 |  |  |  |  |
| 30 | 情爱 | 8 | 幸福 | 56 | 惊奇 | 66 | 欢迎 | 48 | 帮助 | 47 |

Appendix II Top 30 High-Frequency Negative Words in Five Translations

| sequence | | Lin's | frequency | Xu's | frequency | Dong's | frequency | Zhang's | frequency | Zhuang's | frequency |
| --- | --- | --- | --- | --- | --- | --- | --- | --- | --- | --- | --- |
| 1 | 死 | | 98 | 难过 | 112 | 死 | 123 | 死 | 141 | 痛苦 | 174 |
|  | 愕然 | | 33 |  |  |  |  | 难过 | 141 |  |  |
| 2 | 消息 | | 26 | 死 | 106 | 可怕 | 109 | 脾气 | 117 | 死 | 135 |
| 3 | 人为 | | 21 | 不幸 | 101 | 吃惊 | 107 | 害怕 | 83 | 害怕 | 111 |
|  | 徘徊 | | 21 |  |  | 不安 | 107 |  |  |  |  |
| 4 | 不宜 | | 21 | 怀疑 | 93 | 不幸 | 102 | 厉害 | 78 | 惊讶 | 108 |
| 5 | 区区 | | 18 | 惊异 | 92 | 困难 | 100 | 把头 | 76 | 怀疑 | 102 |
|  | 不行 | | 18 |  |  | 悲哀 | 100 |  |  |  |  |
| 6 | 匆匆 | | 18 | 不安 | 76 | 痛苦 | 97 | 消息 | 76 | 卑贱 | 97 |
|  | 不足 | | 16 | 害怕 | 76 |  |  |  |  |  |  |
| 7 | 怏怏 | | 15 | 痛苦 | 71 | 卑贱 | 77 | 痛苦 | 73 | 情绪 | 87 |
| 8 | 泪痕 | | 14 | 悲哀 | 70 | 怀疑 | 75 | 苦恼 | 68 | 难过 | 80 |
|  | 不及 | | 13 |  |  |  |  |  |  | 厉害 | 80 |
| 9 | 小人 | | 12 | 厉害 | 67 | 忍受 | 72 | 不幸 | 66 | 困难 | 78 |
|  | 不悦 | | 11 |  |  | 苦恼 | 72 |  |  |  |  |
| 10 | 模糊 | | 11 | 卑微 | 66 | 陷入 | 66 | 可怕 | 64 | 可怕 | 77 |
| 11 | 不忍 | | 10 | 消息 | 65 | 消息 | 64 | 不对 | 56 | 不幸 | 65 |
|  | 叹息 | | 10 | 可怖 | 65 |  |  | 困难 | 56 | 不行 | 65 |
| 12 | 赫然 | | 10 | 困难 | 63 | 骄傲 | 62 | 眼泪 | 49 | 担心 | 62 |
|  | 无聊 | | 10 |  |  | 难过 | 62 |  |  |  |  |
| 13 | 无知 | | 9 | 眼泪 | 61 | 忧虑 | 54 | 急忙 | 48 | 悄悄 | 59 |
|  | 不适 | | 9 |  |  | 疑问 | 54 |  |  | 眼泪 | 59 |
| 14 | 呜咽 | | 9 | 担心 | 57 | 烦恼 | 51 | 什么的 | 46 | 消息 | 57 |
|  | 悲哽 | | 9 |  |  |  |  |  |  | 倒霉 | 57 |
|  | 寂寞 | | 8 |  |  |  |  |  |  | 不对 | 57 |
| 15 | 无情 | | 8 | 古怪 | 56 | 失去 | 48 | 疑心 | 45 | 忍受 | 55 |
|  | 不易 | | 8 |  |  | 厉害 | 48 |  |  |  |  |
| 16 | 眼泪 | | 8 | 忍受 | 53 | 失望 | 47 | 忍受 | 43 | 失去 | 50 |
|  | 不当 | | 8 | 悄悄 | 53 | 眼泪 | 47 | 对不起 | 43 |  |  |
| 17 | 冒昧 | | 8 | 抛弃 | 52 | 软弱 | 46 | 严厉 | 41 | 对不起 | 49 |
|  | 未必 | | 8 |  |  |  |  | 怀疑 | 41 |  |  |
|  | 罪状 | | 8 |  |  |  |  | 冒昧 | 41 |  |  |
| 18 | 耿耿 | | 7 | 失去 | 47 | 黑暗 | 41 | 伤心 | 40 | 生气 | 48 |
| 19 | 伤心 | | 7 | 卑下 | 44 | 对不起 | 39 | 不定 | 38 | 骄傲 | 43 |
|  | 隐衷 | | 7 |  |  |  |  | 毛病 | 38 |  |  |
|  | 悼亡 | | 7 |  |  |  |  | 呜咽 | 38 |  |  |
| 20 | 无语 | | 7 | 愤怒 | 42 | 罪过 | 38 | 悄悄 | 34 | 讨厌 | 41 |
|  | 怅然 | | 7 |  |  |  |  |  |  | 脾气 | 41 |
|  | 人事 | | 7 |  |  |  |  |  |  | 难以 | 41 |
|  | 丑态 | | 7 |  |  |  |  |  |  | 难受 | 41 |
| 21 | 累累 | | 6 | 严厉 | 40 | 愚蠢 | 37 | 小心 | 33 | 把头 | 39 |
|  | 臃肿 | | 6 |  |  |  |  | 偷偷 | 33 |  |  |
| 22 | 失声 | | 6 | 生气 | 38 | 猜疑 | 36 | 麻烦 | 32 | 随便 | 38 |
|  | 痛哭 | | 6 | 责备 | 38 | 遭遇 | 36 | 愤怒 | 32 | 不好意思 | 38 |
| 23 | 胡来 | | 6 | 沮丧 | 37 | 严厉 | 35 | 后悔 | 31 | 麻烦 | 37 |
|  | 身世 | | 6 |  |  |  |  | 卑鄙 | 31 | 吃惊 | 37 |
|  | 怒容 | | 6 |  |  |  |  | 惊讶 | 31 |  |  |
| 24 | 变故 | | 6 | 对不起 | 34 | 害怕 | 34 | 可笑 | 28 | 紧张 | 35 |
|  | 非人 | | 6 |  |  |  |  | 失望 | 28 | 可笑 | 35 |
|  | 疑虑 | | 6 |  |  |  |  |  |  | 糊涂 | 35 |
| 25 | 为难 | | 6 | 陷入 | 33 | 讨厌 | 33 | 不安 | 27 | 不安 | 34 |
|  | 不肖 | | 6 | 气味 | 33 |  |  | 骄傲 | 27 |  |  |
|  | 卑鄙 | | 5 | 损害 | 33 |  |  | 回头 | 27 |  |  |
|  | 寒微 | | 5 |  |  |  |  | 随便 | 27 |  |  |
|  | 絮絮 | | 5 |  |  |  |  | 厌恶 | 27 |  |  |
| 26 | 畜生 | | 5 | 脾气 | 32 | 愤慨 | 32 | 身后 | 26 | 伤心 | 33 |
|  | 噩梦 | | 5 | 了不得 | 32 | 徘徊 | 32 | 呻吟 | 26 |  |  |
|  | 憔悴 | | 5 |  |  |  |  | 抑郁 | 26 |  |  |
|  | 怒目 | | 5 |  |  |  |  | 小子 | 26 |  |  |
| 27 | 痛楚 | | 5 | 幼稚 | 31 | 模糊 | 31 | 古怪 | 25 | 失望 | 32 |
|  | 麻木 | | 5 |  |  |  |  | 焦灼 | 25 |  |  |
|  | 过时 | | 5 |  |  |  |  | 失去 | 25 |  |  |
|  | 喋喋 | | 5 |  |  |  |  | 大胆 | 25 |  |  |
|  | 狂吠 | | 5 |  |  |  |  | 别扭 | 25 |  |  |
| 28 | 生气 | | 5 | 可怕 | 30 | 疑心 | 30 | 凄凉 | 24 | 伤害 | 31 |
|  | 不得已 | | 5 | 难堪 | 30 |  |  | 屠夫 | 24 | 苦恼 | 31 |
|  | 指斥 | | 5 | 疑心 | 30 |  |  | 阴沉 | 24 |  |  |
|  | 非法 | | 5 | 惊骇 | 30 |  |  |  |  |  |  |
| 29 | 不甘 | | 5 | 阴沉 | 29 | 偷偷 | 29 | 不好意思 | 23 | 身后 | 30 |
|  | 诡秘 | | 5 | 难以 | 29 | 不对 | 29 | 吃惊 | 23 |  |  |
|  | 斜睨 | | 5 | 失望 | 29 | 疯狂 | 29 | 悔恨 | 23 |  |  |
|  | 落漠 | | 5 | 大胆 | 29 | 抛弃 | 29 | 大吃一惊 | 23 |  |  |
|  | 妄语 | | 5 |  |  |  |  | 诧异 | 23 |  |  |
|  | 忧思 | | 5 |  |  |  |  | 怜悯 | 23 |  |  |
| 30 | 羞涩 | | 5 | 丧失 | 28 | 责备 | 28 | 模模糊糊 | 22 | 孤独 | 29 |
|  | 无能 | | 4 | 过分 | 28 | 呜咽 | 28 | 模糊 | 22 | 责怪 | 29 |
|  | 宣泄 | | 4 |  |  | 随便 | 28 | 疑惑 | 22 |  |  |
|  | 小子 | | 4 |  |  |  |  | 讨厌 | 22 |  |  |
|  | 不幸 | | 4 |  |  |  |  | 死气白赖 | 22 |  |  |
|  | 皱纹 | | 4 |  |  |  |  | 担心 | 22 |  |  |

Appendix III Comparison of emotion types in Martin & White (2005) and in the Chinese Dictionary

| emotion sets | positive | happiness | 喜欢 | PB +5 | 好 (goodness) |
| --- | --- | --- | --- | --- | --- |
|  |  |  | 高兴 | PA +5 | 乐  （joy） |
|  |  |  | 快乐 | PA +5 |  |
|  |  |  | 愉快 | PA +5 |  |
|  |  |  | 快活 | PA +7 |  |
|  |  |  | 朋友 | PB +9 |  |
|  |  | security | 相信 | PG +7 | 好  (goodness) |
|  |  |  | 一定 | PG +5 |  |
|  |  |  | 必须 | PG +7 |  |
|  |  |  | 肯定 | PG +3 |  |
|  |  |  | 朋友 | PB +9 |  |
|  |  | satisfaction | 不错 | PH +3 | 好  (goodness) |
|  |  |  | 可爱 | PH +5 |  |
|  |  |  | 漂亮 | PH +6 |  |
|  |  |  | 神气 | PH +5 |  |
|  |  |  | 朋友 | PB +9 |  |
|  |  | inclination | 希望 | PK +5 | 好(goodness) |
|  |  |  | 愿意 | PA +1 | 乐（joy） |
|  | negative | unhappiness | 难过 | NB -5 | 哀  (sadness) |
|  |  |  | 痛苦 | NB -7 |  |
|  |  |  | 眼泪 | NB -5 |  |
|  |  |  | 不幸 | NB -9 |  |
|  |  |  | 悲哀 | NB -5 |  |
|  |  |  | 苦难 | NB -5 |  |
|  |  |  | 忍受 | NI -9 | 惧（fear） |
|  |  |  | 死 | NX -7 |  |
|  |  | insecurity | 消息 | NF-1 | 哀 (sadness) |
|  |  |  | 怀疑 | NL -9 | 恶 (disgust) |
|  |  |  | 惊异 | NC -5 | 惧 (fear) |
|  |  |  | 吃惊 | NC -5 |  |
|  |  |  | 惊讶 | NC -5 |  |
|  |  |  | 不安 | NI -5 |  |
|  |  |  | 死 | NX -7 |  |
|  |  | dissatisfaction | 卑微 | NN -1 | 恶 (disgust) |
|  |  |  | 卑贱 | NN -9 |  |
|  |  |  | 苦恼 | NE -5 |  |
|  |  |  | 陷入 | NE -3 |  |
|  |  |  | 情绪 | NE -5 |  |
|  |  |  | 脾气 | NA -5 | 怒 (anger) |
|  |  |  | 死 | NX -7 |  |
|  |  | disinclination | 害怕 | NC -3 | 惧 (fear) |
|  |  |  | 厉害 | NC -3 |  |
|  |  |  | 可怕 | NC -3 |  |
|  |  |  | 死 | NX -7 |  |
